# Deployment of the National Notifiable Diseases Surveillance System during the 2022–23 mpox outbreak in the United States—Opportunities and challenges with case notifications during public health emergencies

**DOI:** 10.1371/journal.pone.0300175

**Published:** 2024-04-11

**Authors:** Jeanette J. Rainey, Xia Michelle Lin, Sylvia Murphy, Raquel Velazquez-Kronen, Tuyen Do, Christine Hughes, Aaron M. Harris, Aaron Maitland, Adi V. Gundlapalli

**Affiliations:** 1 Division of Global Health Security, Global Health Center, Centers for Disease Control and Prevention, Atlanta, GA, United States of America; 2 Detect and Monitor Division, Office of Public Health Data, Surveillance, and Technology, Centers for Disease Control and Prevention, Atlanta, GA, United States of America; 3 Division of Field Studies and Engineering, National Institute for Occupational Safety and Health, Centers for Disease Control and Prevention, Cincinnati, OH, United States of America; 4 Office of the Director, National Center for Emerging and Zoonotic Diseases, Centers for Disease Control and Prevention, Atlanta, GA, United States of America; 5 Division of High-Consequence Pathogens and Pathology, National Center for Emerging and Zoonotic Diseases, Centers for Disease Control and Prevention, Atlanta, GA, United States of America; 6 Division of Health Interview Statistics, National Center of Health Statistics, Centers for Disease Control and Prevention, Hyattsville, MD, United States of America; 7 Office of the Director, Office of Public Health Data, Surveillance, and Technology, Centers for Disease Control and Prevention, Atlanta, GA, United States of America; Tehran University of Medical Sciences, ISLAMIC REPUBLIC OF IRAN

## Abstract

Timely case notifications following the introduction of an uncommon pathogen, such as mpox, are critical for understanding disease transmission and for developing and implementing effective mitigation strategies. When Massachusetts public health officials notified the Centers for Disease Control and Prevention (CDC) about a confirmed orthopoxvirus case on May 17, 2023, which was later confirmed as mpox at CDC, mpox was not a nationally notifiable disease. Because existing processes for new data collections through the National Notifiable Disease Surveillance System were not well suited for implementation during emergency responses at the time of the mpox outbreak, several interim notification approaches were established to capture case data. These interim approaches were successful in generating daily case counts, monitoring disease transmission, and identifying high-risk populations. However, the approaches also required several data collection approvals by the federal government and the Council for State and Territorial Epidemiologists, the use of four different case report forms, and the establishment of complex data management and validation processes involving data element mapping and record-level de-duplication steps. We summarize lessons learned from these interim approaches to inform and improve case notifications during future outbreaks. These lessons reinforce CDC’s Data Modernization Initiative to work in close collaboration with state, territorial, and local public health departments to strengthen case-based surveillance prior to the next public health emergency.

## Introduction

Mpox (formerly known as monkeypox) is a zoonotic disease caused by the Monkeypox virus, an Orthopoxvirus (OPV) in the Poxviridae family. The virus is endemic in Central and West Africa. Prior to 2022, the introduction of the virus to non-endemic regions occurred through the importation of infected animals or sick travelers [1, 2]. Transmission can occur through close contact with rashes, lesions, or bodily fluids from persons or animals with mpox [3–5]. During the 2022–23 outbreak in the United States, transmission occurred primarily through sexual contact [1, 6, 7], which differed from previous human mpox outbreaks. Laboratory confirmation relies on real-time polymerase chain reaction (PCR) assays for the detection of orthopoxvirus (including non-variola-specific orthopoxvirus) or Monkeypox virus from lesion specimens [8, 9]. Primary mitigation measures include isolation of patients and contact tracing, vaccine delivery to high-risk populations, and dissemination of public health messages [10, 11].

When Massachusetts public health officials notified the Centers for Disease Control and Prevention (CDC) about a confirmed orthopoxvirus case on May 17, 2023, which was later confirmed as mpox, mpox was not a nationally notifiable disease [1, 6]. Although several state, territorial, and local public health departments, hereafter referred to as STLPHDs, had mandated mpox case reporting, no approved systematic data collection processes were available for mpox case notifications at the national level. As a result, CDC implemented several interim case notifications approaches during the mpox outbreak. These case data were critical for tracking the outbreak, identifying high-risk groups, and ensuring public health equity [12–14].

STLPHDs—50 states, New York City, Washington DC, 5 territories, and 3 freely associated states (**S1 Appendix. List of STLPHDs**)—and the Council of State and Territorial Epidemiologists (CSTE) work closely with CDC to establish procedures and policies for sending case notifications through the National Notifiable Diseases Surveillance System (NNDSS) [15]. STLPHDs can submit core data elements (same data elements for all diseases and conditions) and disease-specific data elements to CDC through the following formats: the Health Level Seven (HL7) Case Notification Message [16], the National Electronic Telecommunications System for Surveillance (NETSS), and the National Electronics Disease Surveillance System (NEDSS) Base System (NBS) Master Message [17]. At CDC, case data are processed in the Message Validation, Processing, and Provisioning System (MVPS) and provisioned to disease-specific programs at CDC. Data can also be provisioned to the Data Collection and Integration for Public Health Event Response (DCIPHER) platform, a cloud-based data management platform rolled-out in 2020 to support emergency response and used extensively during the national COVID-19 pandemic response [18].

Current public health informatics infrastructure, including NNDSS, however, lacks the flexibility and scalability needed to meet the informational needs following the introduction of a novel disease or a rapidly evolving outbreak of a non-notifiable disease [19, 20]. Collecting data specific to a novel pathogen, for example, can require developing and implementing disease-specific Message Mapping Guides (MMGs), which involves message design and development, incorporation into MVPS, user acceptance assessment, and publication of final MMGs [16]. To help address these limitations, CDC launched the Data Modernization Initiative (DMI) in 2020 [21]. DMI aims to protect the United States population from any health threat, including the introduction of a novel or re-emerging pathogen such as mpox, by efficiently collecting high-quality and actionable data for decision-making at all levels of public health. In this paper, we describe the use of interim approaches for case notifications during the mpox outbreak, including challenges associated with increasing case counts and changes in case notification requirements for STLPHDs. Our findings highlight the importance of implementing DMI’s ‘response ready’ surveillance goal prior to the next public health emergency [21].

## Methods

### Initial case notifications via the Call Center and REDCap

**Fig 1:**
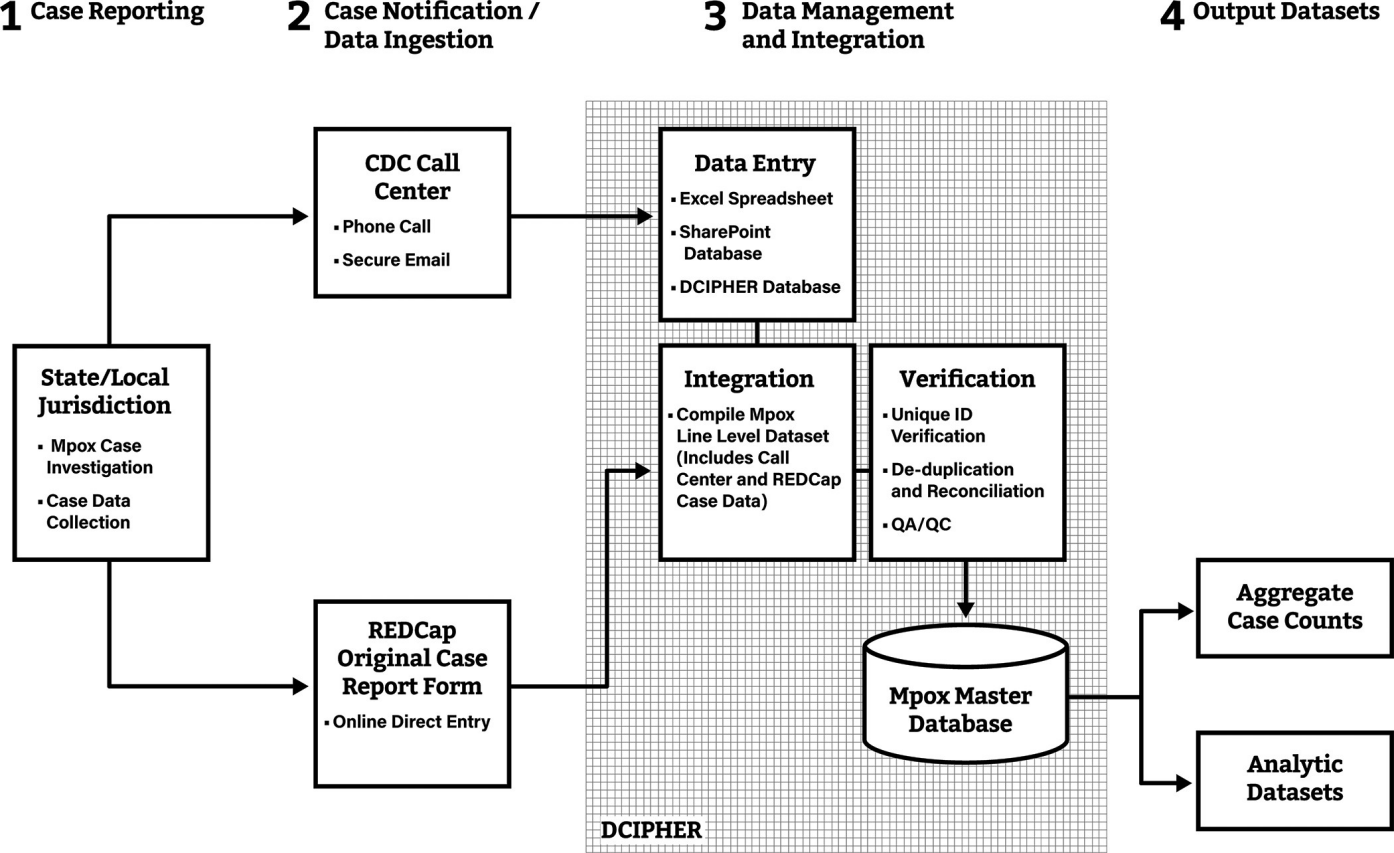
Data flow of mpox case notifications from STLPHDs to CDC through the Call Center and REDCap, May 17, 2022 to July 15, 2022*. *From May 17 to July 15, 2022, STLPHDs sent mpox case notifications to CDC’s Call Center; initially, CDC Call Center staff entered mpox case data into an Excel spreadsheet, later into a SharePoint database, and then directly into the Data Collation and Integration for Public Health Event Response (DCIPHER) platform. STLPHDs could send additional case data using the original case report form (CRF) via REDCap (a secure web-based application used to support data collection activities). CDC and STLPHDs revised approaches for assigning unique IDs for case notifications several times during the first weeks of the outbreak. Verifying unique IDs for each case was required before de-duplication, record reconciliation, and generating a Master mpox database.

Following reports of mpox from several STLPHDs in May 2022, CDC rapidly established a Call Center to provide guidance for STLPHDs investigating suspect mpox cases. CDC requested STLPHDs to share information on suspect, probable, and confirmed mpox cases to CDC’s Call Center staff by phone or through a secure email exchange. CDC’s Call Center staff entered de-identified case data received from STLPHDs into an Excel spreadsheet for the first few days of the response (**Table 1 and Fig 1**). This process was transitioned to a Microsoft PowerApp (Microsoft Corporation, Redmond, WA) with a SharePoint database backend. CDC staff manually created unique case identification numbers (IDs) for each suspect case; laboratory test results from the Laboratory Response Network [22] were added to case data according to case ID. Call Center staff also entered information on sex at birth, age, symptom onset date (i.e., fever and rash), place of residence, travel history, and sexual orientation and gender identity (SOGI) when available and provided by STLPHDs [7].

**Table 1:** Mpox case notification approaches and corresponding start and end dates, May 17, 2022 to January 31, 2023*.

Approach	Start Date	End Date
Call Center	5/17/2022	7/15/2022
REDCap—Original CRF	6/3/2022	7/15/2022
Short CRF via DCIPHER	7/1/2022	Ongoing
NNDSS Core Data	7/23/2022	Ongoing

*CRF–Case Report From; DCIPHER–Data Collection and Integration for Public Health Event Emergencies

platform; NNDSS–National Notifiable Diseases Surveillance System.

SharePoint case data were later uploaded to a DCIPHER database. Following feedback from STLPHDs, only probable and confirmed case notifications were sent to CDC. By the end of June 2022, Call Center staff were entering data on probable and confirmed case notifications received from STLPHDs directly into DCIPHER; unique case IDs were automatically generated in DCIPHER. Case notifications received by the Call Center by 2:00 pm each day were included in CDC’s official daily case counts for national-level situational awareness and public reports. Case notifications received after 2:00 pm were added to CDC’s case counts for the following day.

On June 3, 2022, CSTE provided guidance to STLPHDs on sending mpox case notifications to CDC through REDCap. REDCap is a secure, Health Insurance Portability and Accountability Act (HIPAA) compliant, web-based application designed to support data collection for research studies and surveillance activities [23]. The original mpox CRF, accessible via REDCap and hosted at CDC, collected information on 223 demographic, clinical, exposure, laboratory testing date and result variables. CDC initially relied on outbreak response authority for collecting case data from STLPHDs and later received emergency approval for the use of the original mpox CRF, including for data elements on SOGI, from the United States Office of Management and Budget (OMB), as part of the 1972 Paper Reduction Act [24]. STLPHDs were able to manually enter case data to REDCap according to the STLPHDs’ unique case identification number, if available. CSTE added mpox as a nationally notifiable disease on June 23, 2022 [25]. STLPHDs contacted the Call Center for initial case notifications and were able to provide additional case data via REDCap. CDC requested STLPHDs to send case notifications through alternative approaches on July 1. Due to the large volume of cases occurring across the country and availability of other pathways for sharing case data, the Call Center and REDCap were closed for case notifications on July 15, 2023.

### Roll-out of short case report form

**Fig 2:**
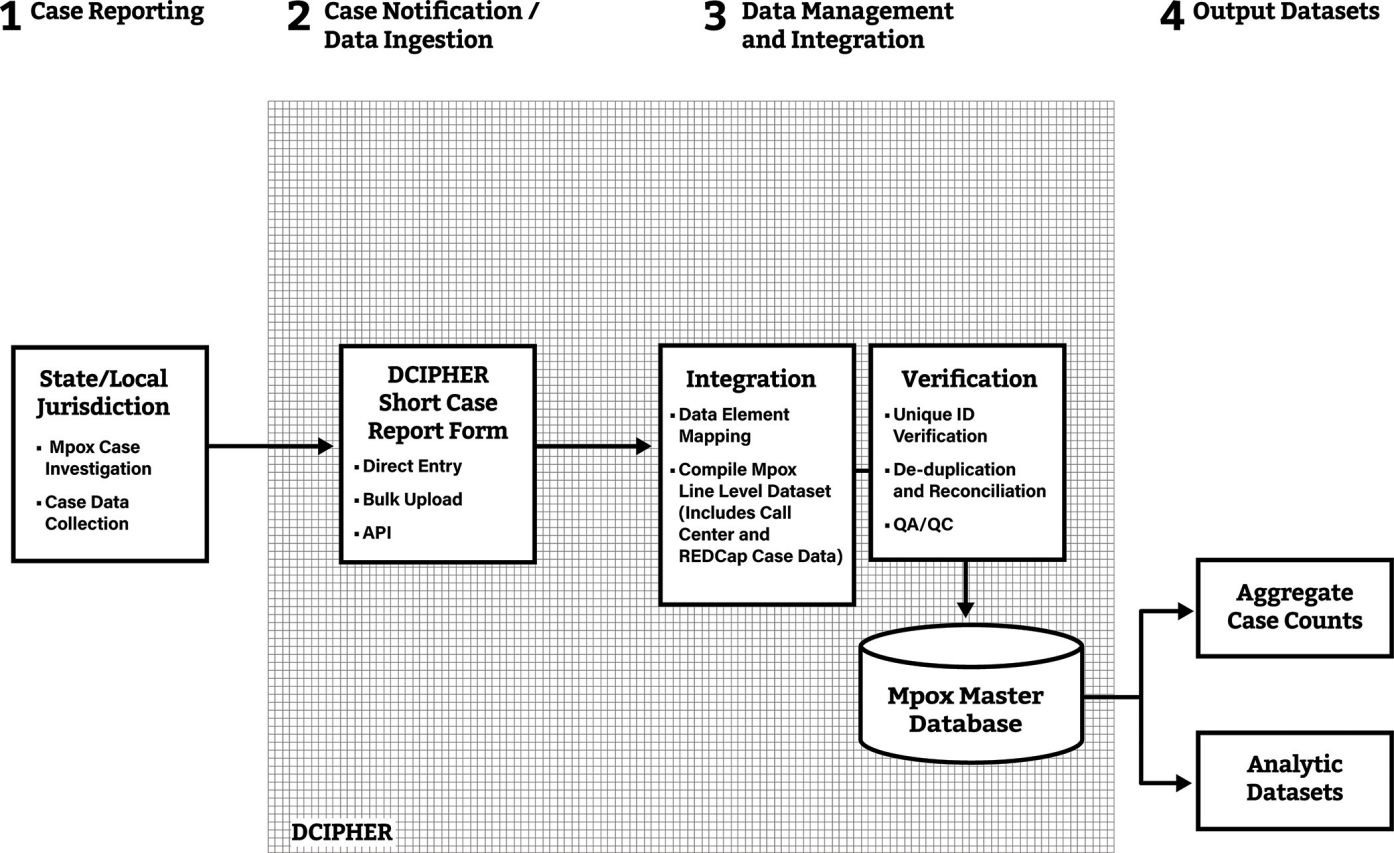
Data flow of mpox case notifications from STLPHDs to CDC through DCIPHER using the short CRF, July 1, 2022 to July 23, 2022*. *After roll-out of the short case report from (CFR) on July 1, 2022, STLPHDs sent mpox case notifications directly to the Data Collation and Integration for Public Health Event Response (DCIPHER) platform. Data elements from the Call Center and REDCap were mapped to the short CRF. A Master mpox database was generated by compiling, verifying, de-duplicating, and reconciling records previously received from the Call Center, REDCap, and DCIPHER, using the data elements included in the short CRF. API refers to Application Programming Interface, an option for STLPHDs to send case data to DCIPHER.

On July 1, 2022, CDC received expedited OMB approval and released the short CRF for mpox case notifications. The short form collected 23 data elements. STLPHDs could send mpox case notifications manually by entering short CRF data directly in CDC’s DCIPHER platform or by entering the necessary data into their local disease surveillance system configured for mpox, and then uploading case data in bulk to DCIPHER using a CSV file format (**Fig 2**). STLPHDs were also able to send case notifications using the short CRF via an Application Programming Interface (API) with appropriate authentication and security. Case notifications were sent using a STLPHD-assigned unique ID. CDC staff mapped data elements captured from the Call Center and REDCap to the short CRF. CDC collaborated with STLPHDs to reconcile mpox case notifications (i.e., obtaining various STLPHD-assigned unique record IDs) from the Call Center, REDCap, and short CRF to generate a Master mpox database in DCIPHER. If duplicate case records with the same STLPHD case ID were detected following reconciliation, a pre-defined algorithm was applied to select the record with the most complete mpox case data.

Because of initial vaccine supply limitations, case notifications sent to CDC using the short CRF were used to estimate the allocation of the national stockpile of JYNNEOS to STLPHDs. Vaccine allocation involved population-based weighting and case-based weighting; the latter of which relied on the cumulative number of case notifications received from each STLPHD on June 28 (Phase 1), July 6 (Phase 2A), July 15 (Phase 2B), and lastly on July 27 (Phase 3). Population-based weighting reflected the at-risk groups associated with the mpox outbreak, primarily men-who-have-sex-with-men (MSM), and case-based weighting targeted current transmission hotspots.

### Mpox case notifications via NNDSS

**Fig 3:**
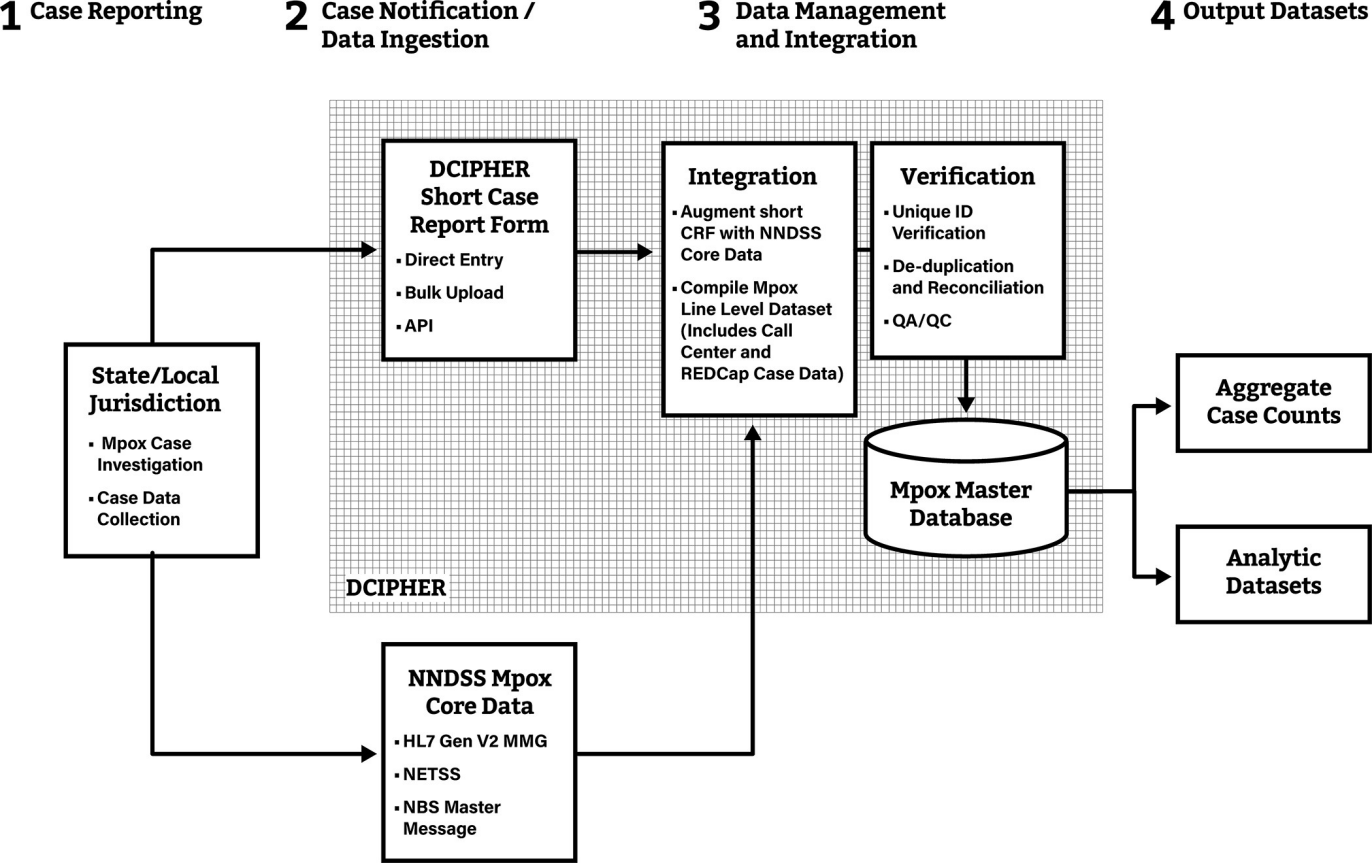
Data flow of mpox case notifications from STLPHDs to CDC through NNDSS and DCIPHER, from July 23, 2022 onwards*. *CDC onboarded STLPHDs to the National Notifiable Diseases Surveillance System (NNDSS) on July 23, 2022. STLPHDs sent core mpox data to NNDSS using HL7 message mapping (MMG) Gen v2, NETSS, and NBS Master Message. STLPHDs sent mpox specific data using the short case report form (CRF) via the Data Collation and Integration for Public Health Event Response (DCIPHER) platform. NNDSS core data were augmented with short CRF data in DCIPHER. A Master mpox case database was generated by compiling, verifying, de-duplicating, and reconciling records previously received by the Call Center, REDCap, and DCIPHER. API refers to Application Programming Interface, an option for STLPHDs to send case data to DCIPHER.

On July 23, 2023, CDC received expedited OMB approval to receive mpox case notifications from STLPHDs through NNDSS. STLPHDs submitted formal requests to CDC prior to implementing the NNDSS onboarding process. NNDSS core data were mapped to the corresponding data elements in the short CRF. By default, NNDSS data values had higher priority if case notifications had values for the same data element from both sources. STLPHDs, however, had the option of selecting specific data element values from the short CRF and overwriting NNDSS values within DCIPHER. As with other nationally notifiable diseases, core surveillance data elements were submitted using previously established formats for HL7 Case Notification Message, NETSS, or NBS master message (**Fig 3**). HL7 messages following the generic (version 2) message mapping guide (Gen v2 MMG) were preferred, but legacy Gen v1 MMG, NETSS data and NBS master messages were also allowed. STLPHDs could continue to send mpox case data using the short CRF, directly to DCIPHER through manual entry, bulk uploads, or via an API. Following validation and finalizing data stream pipelines, case notifications sent through NNDSS were augmented with case data sent to DCIPHER.

Supplemental funding was provided to STLPHDs through CDC’s Mpox Crisis cooperative agreement. Initial funding was awarded to STLPHDs according to the number of mpox notifications sent to CDC in October 2022 with an illness onset date of October 2022 (or the closest approximate when illness onset date was not available) [14]. Award amounts reflected estimated response needs based on the disease burden in October 2022.

Mpox case notifications sent to CDC adhered to federal policies and regulations on data collections for new diseases and conditions [24] and did not include personal identifying information. The authors of this paper were involved in various aspects of receiving, verifying, and analyzing case notifications during the mpox outbreak. CDC’s Office of the Associate Director of Science in the Emergency Operations Center reviewed and approved this project as a surveillance activity, consistent with CDC policy and applicable federal law. Because this project relied on national case notifications, consent from case-patients was waived. Aggregate mpox case counts and case counts by select demographics were publicly reported and updated on the CDC mpox website on a regular basis throughout the outbreak. The United States declared the mpox outbreak a public health emergency on August 4, 2022 [26]. The public health emergency was declared over on January 31, 2023.

## Results

**Fig 4:**
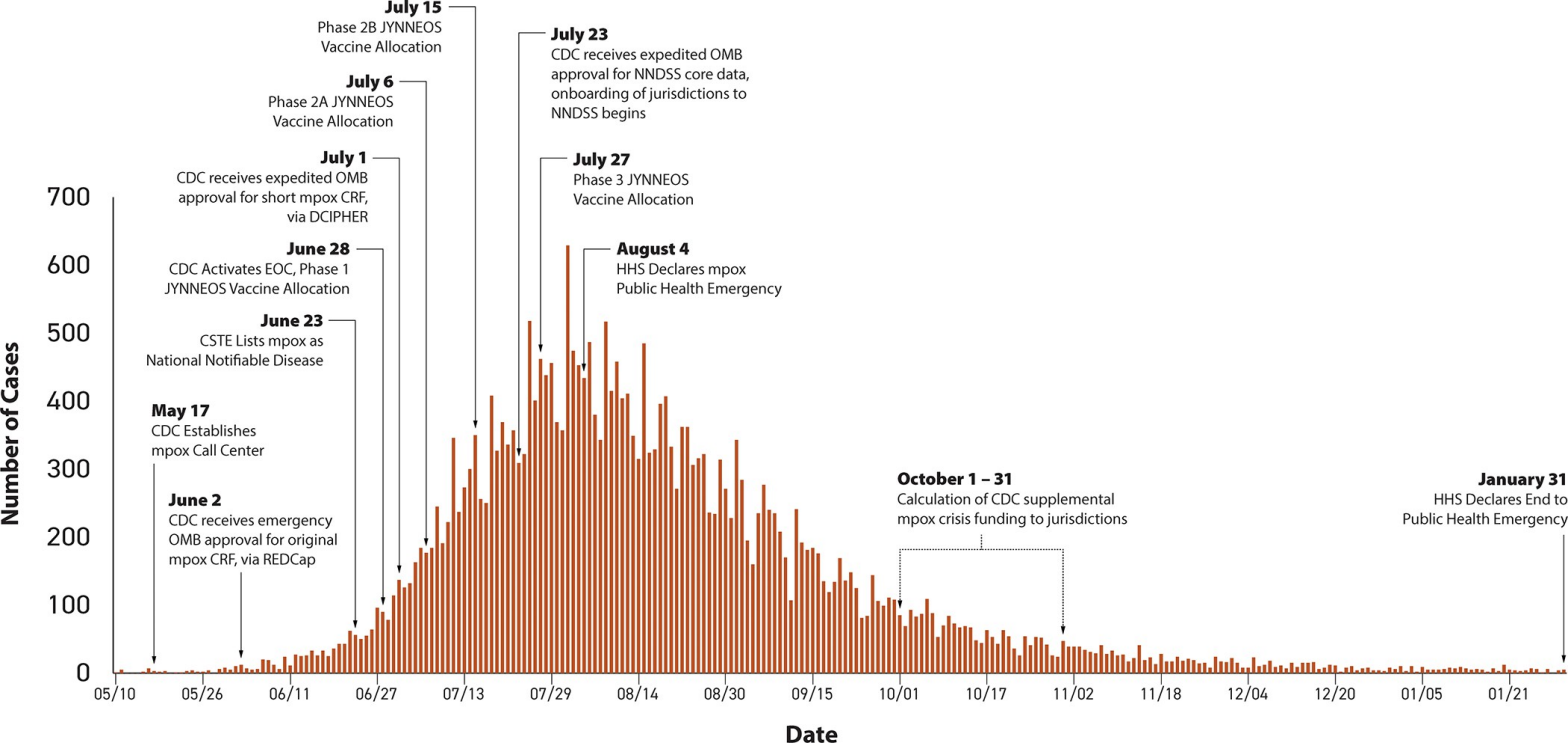
Epidemiologic curve of mpox case notifications, May 10, 2022, to January 31, 2023. Interim case notification approaches are noted by implementation date*. *Daily case counts obtained from the Centers for Disease and Control and Prevention’s official aggregate mpox case count dataset. Available at: https://www.cdc.gov/poxvirus/mpox/response/2022/mpx-trends.html.

Between May 17, 2022, and January 31, 2023, CDC received 30,157 mpox case notifications from 54 STLPHDs; 50 states plus Puerto Rico, Washington DC, New York City, and Philadelphia (**Fig 4**). Case notifications from New York City and Philadelphia were included in official daily case counts for the States of New York and Pennsylvania, respectively.

### Initial notifications via the Call Center and REDCap

Between May 17 to June 23, CDC’s Call Center received a mean of 14 (range: 1–62) mpox case notifications per day. This increased to 77 per day (range: 49–111) during the last week of June. Twenty-one STLPHDs sent mpox case data using the original CRF via REDCap during this same time frame.

### Roll-out of short case report form

After roll-out of the short CRF on July 1, most (n = 32, 60%) STLPHDs sent mpox case notifications to DCIPHER using a combination of direct-data entry, bulk uploads, and APIs. Another 19 (35%) STLPHDs used direct-data entry to DCIPHER as the single approach for sending short CRF data to CDC, and the remaining 4 (20%) used bulk uploads only. Daily case counts peaked on August 1, 2022, with a total of 629 mpox case notifications. Sixty-three STLPHDs were eligible to receive an allocation of JYNNEOS vaccine, including two counties and one city whose mpox case reports were sent to CDC as part of a state’s outbreak-related case notifications. According to available data, 31 STLPHDs received an allocation of JYNNEOS vaccine during Phase 1, 62 and 61 during Phase 2A and 2B, and 62 during Phase 3.

### Mpox case notifications via NNDSS

Between July 23, 2022, and January 31, 2023, 54 STLPHDs sent mpox data to DCIPHER using the short CRF, and 17 (31%) of these STLPHDs also sent core case data to NNDSS; 15 via HL7 Gen v2 MMG and 2 via NETSS. Of these 17 STLPHDs, six were onboarded to NNDSS between July and October 2022, another six during November and December 2022, and the remaining five by the end of January 2023.

A total of 43 STLPHDs sent case notifications during October 2022 to CDC with an October mpox illness onset date (or close approximate), corresponding to the supplemental funding request. The number of case notifications from these 43 STLPHDs ranged from 1 to 301 (median: 21). In December 2022, CDC awarded mpox response funding to 21 STLPHDs (11 state health departments and 10 local health departments—nine whose mpox case reports were sent to CDC as part of a state’s outbreak-related case notifications).

## Discussion

Due to limitations with existing NNDSS processes for rapidly deploying new data collections, CDC implemented several interim mpox case notification approaches. These approaches were successfully used to track national case counts, identify high-risk populations to support public health equity [12], and allocate JYENNOS vaccine [13] and supplemental funding to STLPHDs [14]. Reliance on these interim approaches, however, affected the efficiency of mpox case notifications, particularly related to the ingestion, management, and validation of mpox data, and created additional burden for STLPHDs as well as for CDC response staff (**Table 2**). Deployment of these interim approaches and challenges in onboarding STLPHDs to NNDSS further inform and reinforce CDC’s Data Modernization Initiative [21].

**Table 2:** Summary of challenges and proposed solutions from the deployment of interim case notification approaches during the mpox outbreak in the United States, May 17, 2022–January 31, 2023.

Challenge	Proposed DMI Solution
**Data collection authority** OMB Paperwork Reduction Act requirements for new data collections Lack of data use/sharing agreements	Exempt OMB Paperwork Reduction Act requirements for case notifications on emerging diseases or conditions for a declared public health emergency or in support of the CDC Emergency Operations Center for an activated agency-wide response. Establish high level data use agreements in advance between CDC and STLPHDs for multi-state outbreak situations and/or introduction of novel infections (e.g., SARS-CoV2) or identification of new risk groups (mpox).
**Data Ingestion** Extended/unrealistic reliance on Call Center for case notifications Lack of system interoperability between CDC and local STLPHDs	Establish trigger or threshold for early transition from Call Center to established/sustainable case notification procedures for emergency situations. Streamline processes and increase interoperability by supporting additional/format-flexible data transfer processes beyond HL7 message mapping, allowing CDC to receive case notifications in multiple formats and from multiple sources, depending on existing systems and capabilities at each STLPHD.
**Data Management** Lack of flexibility in adding new data elements to recognize and address minority at-risk populations (i.e., health equity) Multiple unique IDs for the same case records	Create and verify a list of data elements that adhere to CDC’s data standards, including for non-notifiable diseases and conditions that can be pre-approved by OMB and available when needed for a new outbreak-related data collection. Create a cloud-based ’scalable’ approach to add disease-specific data elements from a pre-approved list, as needed to address public health equity during an emergency response. Establish process to ensure that all unique case records can be easily identified, using either a STLPHD assigned ID or a combination of data elements sent with each case notification, based on agreements between STLPHDs, CSTE, and CDC in advance of the next outbreak.
**Data Integration** Use of several data entry points for sending case notifications to CDC (e.g., NNDSS core data and DCIPHER for mpox specific data) High level of burden on STLPHDs, with limited perceived benefits	Create "One Front Door" at CDC for case notifications where STLPHDs can send all case data to the CDC using a single-entry point, regardless of disease or condition or data format—or use of a Public Health Data Router where STLPHD case data flow to a single point at CDC and the router properly routes data to the right system. Provide technical support and resources to STLPHDs to upgrade local surveillance systems.

New data collections, including during a public health emergency, require OMB review and approval. Although intended to minimize the potential burden on individuals, businesses, and state and local STLPHDs [24], this requirement can unintentionally delay the request for new case notifications and additional data elements. An umbrella OMB package for emergency responses exists and was used to collect data for mpox case notifications. During the outbreak, however, OMB review and approval was also required for data elements collected in the original CRF via REDCap, changes in the data elements collected in the short CRF sent to DCIPHER, and later for the core data elements captured through NNDSS. Proposed solutions include exempting Paperwork Reduction Act requirements for case notifications on emerging diseases or conditions for a declared public health emergency or in support of the CDC Emergency Operations Center for an activated agency-wide response. Such solutions—along with high-level data use agreements between STLPHDs and CDC—could allow public health officials to better prepare for and respond to the need for emergency case notifications in the future.

The original purpose of CDC’s Call Center was to provide STLPHDs technical advice about diagnostic testing and case management [1, 6]. During the early weeks of the mpox outbreak, the Call Center also received case notifications by email or phone, which were manually entered into an Excel spreadsheet, SharePoint database, and then into DCIPHER. The Call Center was a short-term workable solution but was not intended to be scalable for a large outbreak. By the fifth week of the mpox outbreak, the Call Center was receiving more than 77 case notifications per day from more than a dozen STLPHDs. This case load significantly affected the ability of CDC staff to review and verify case data in a timely manner. The mpox outbreak was declared a public health emergency in the United States only on August 4, 2022 [26], after the daily case count peaked on August 1 with 629 mpox case notifications. In the future, possible triggers for transitioning to a sustainable case notification process for a new non-notifiable disease could include evidence of ongoing transmission, such as two generations of the new disease in a single STLPHD or two generations epidemiologically linked across two or more STLPHDs. Establishing an a-priori trigger could increase efficiencies in the ingestion of case notifications required to identify high-risk populations and monitor transmission trends, while allowing the Call Center to focus on addressing clinical and laboratory diagnostic inquiries from STLPHDs. Such an approach could also facilitate the use of case notifications for allocating vaccine and other medical countermeasures according to local need and disease burden.

De-duplication and reconciliation of mpox case notifications across the interim approaches required complex data management and validation processes. Although unique case IDs were requested on the original and short CRF, most STLPHDs did not have formal systems in place for collecting mpox data, and the evolving outbreak necessitated changing the process of assigning case IDs over time. As a result, the reconciliation process was time- and labor-intensive and required additional work by many STLPHDs and CDC staff. As part of DMI, CDC could work with CSTE and STLPHDs to proactively establish a process of assigning unique case IDs during a large outbreak or public health emergency. For routine surveillance of nationally notifiable conditions through NNDSS, two data elements—locally assigned record ID and the name of the STLPHD—are used to identify unique cases for HL7 case notification messages transmitted to MVPS; four data elements are used for NETSS. Establishing a standard process in advance could reduce the data management burden to reconcile and de-deduplicate records during an outbreak or emergency response.

During the initial months of the mpox outbreak, four different case notification tools or forms were used: Call Center SharePoint site, original mpox CRF via REDCap, short CRF through DCIPHER, and core data elements submitted through NNDSS. Although the use of different data collection forms was necessary during early stage of the mpox outbreak, transition between these forms required CDC to map data elements across the forms, temporarily impacting CDC’s ability to generate up-to-date epidemiologic output [7, 27]. To prepare for the next large outbreak or pandemic, CDC could work with CSTE to identify feasible approaches to minimize these challenges. For example, in collaboration with CSTE, a list of data elements adhering to national data standards could be developed—from nationally notifiable and non-notifiable conditions across various disease etiologies, modes of transmission, and clinical considerations and shared with STLPHDs. Case notifications requested early during public health emergencies could pull from this single list of data elements. Scalable disease-specific CRFs could be established within a cloud-based environment, allowing flexibility to add (or remove) elements—meeting CDC’s and OMB approved data standards—to address disease specific risk factors, public health equity (e.g., gender identity) [12], and feedback from STLPHDs. This could help reduce the need for different CRFs and data mapping across CRFs during the early critical phases of an outbreak, thereby increasing the ability to rapidly analyze case notification data for time-sensitive decision making.

Current processes for establishing new data collections within NNDSS are unlikely to meet the timeliness requirements during outbreak scenarios, particularly for non-standard data elements which may be required in response to a novel or re-emerging infectious disease outbreak. Streamlining processes and increasing interoperability by supporting format-flexible data transfer processes beyond HL7 message mapping would likely facilitate new outbreak-related data collections [16]. Although NNDSS was available for mpox case notifications in late July, disease-specific data were sent separately to DCIPHER using the short CRF, resulting in double reporting processes using two different case notification entry-points. In the future, CDC could leverage a “One Front Door” approach where STLPHDs are able to send all case data—core data and supplemental disease-specific data—to the CDC using a single-entry point. Other options include the use of a public health data router, where STLPHD case data flows to a single point at the CDC, and the router properly routes data to the right system. Both “One Front Door” options would eliminate the need for STLPHDs to send data to multiple sites or locations. New format-flexible processes would allow STLPHDs to send case data using their existing surveillance infrastructure and capabilities. These options could substantially improve CDC’s capacity to rapidly distribute outbreak-related resources using an equitable process to address existing disease burden and potential disease-risk.

Several limitations were identified during this project. First, the challenges described in this paper reflect informal feedback received from CSTE and various STLPHDs during the mpox outbreak. CDC’s mpox response team worked closely with CSTE and STLPHDs during the outbreak to learn from and address concerns with the interim case notification approaches. However, a separate formal follow-up survey or focus group discussions may have elicited more detailed feedback. For example, only 17 of 54 STLPHDs sent mpox core data using NNDSS. Whether this was due to a lack of interoperability with NNDSS HL7 Gen v2 or other issues is not clear. Second, we were unable to assess the impact of the interim approaches on the timeliness and completeness of the case notifications sent to CDC. Based on epidemiologic data analysis during the outbreak, completeness of case notifications appeared to increase overtime [7, 28]. Because of the variability across STLPHDs and concurrent use of several of the interim approaches, an in-depth analysis of case data in relation to each case notification approach was deemed beyond the scope of this project. Finally, we were unable to determine the extent to which the COVID-19 pandemic influenced the roll-out, acceptance, and use of the interim case notification approaches, particularly in high-risk STLPHDs [29–31]. While the use of new tools and platforms such as DCIPHER were likely beneficial [20], the request for mpox case notifications–along with COVID-19 data—likely created additional burden for many STLPHDs [32–34]. CDC’s DMI will need to reduce the burden on STLPHDs for sending case notifications by streamlining data ingestion flows and minimizing—or automating—internal data management, validation, and integration steps [21].

## Conclusion

Because NNDSS was not developed to support new outbreak related data collections, several interim case notification approaches were deployed during the mpox outbreak in 2022. These approaches allowed CDC to obtain critical case data to track the outbreak, identify high-risk groups, and allocate vaccine and supplemental funding to STLPHDs. Deployment of these interim approaches, however, involved several challenges related to case data ingestion, management, and integration. Proposed solutions include establishment of a scalable “One Front Door” where STLPHDs can send all case data to CDC using a single-entry point or public health router, development of format agnostic (i.e., format-free) data transfer processes, and investments to strengthen local and surveillance infrastructure. Regulatory modifications related to new data collections are also needed, allowing CDC to work efficiently with CSTE and STLPHDs to respond to emergencies with real-time case notifications as soon as a novel disease or emerging outbreak is recognized. Our project reinforces the importance of DMI and CDC’s goal of a response-ready surveillance system.

## Supporting information

S1 Appendix List of STLPHDs. (DOCX)
