# Supplementary material for: Deployment of the National Notifiable Diseases Surveillance System during the 2022–23 mpox outbreak in the United States—Opportunities and challenges with case notifications during public health emergencies
Source: PLoS One. 2024 Apr 11;19(4):e0300175. doi: 10.1371/journal.pone.0300175 (PMC11008850; doi:10.1371/journal.pone.0300175)
Supplement: S1 Appendix — (DOCX) [file pone.0300175.s001.docx]

**S1 Appendix - List of 60 State, Territories, and Local Public Health Departments sending case notifications to the Centers for Disease Control and Prevention**

States:

Alabama

Alaska

Arizona

Arkansas

California

Colorado

Connecticut

Delaware

Florida

Georgia

Hawaii

Idaho

Illinois

Indiana

Iowa

Kansas

Kentucky

Louisiana

Maine

Maryland

Massachusetts

Michigan

Minnesota

Mississippi

Missouri

Montana

Nebraska

Nevada

New Hampshire

New Jersey

New Mexico

New York

North Carolina

North Dakota

Ohio

Oklahoma

Oregon

Pennsylvania

Rhode Island

South Carolina

South Dakota

Tennessee

Texas

Utah

Vermont

Virginia

Washington

West Virginia

Wisconsin

Wyoming

Federal District

District of Columbia

Local Public Health Department

New York City

Territories

American Samoa

The Commonwealth of Northern Mariana Islands

Guam

Puerto Rico

United States Virgin Islands

Freely Associated States

The Federated States of Micronesia

The Republic of the Marshall Islands

The Republic of Palau
